# Identification of long non-coding RNAs expressed in knee and hip osteoarthritic cartilage

**DOI:** 10.1016/j.joca.2018.12.015

**Published:** 2019-04

**Authors:** B. Ajekigbe, K. Cheung, Y. Xu, A.J. Skelton, A. Panagiotopoulos, J. Soul, T.E. Hardingham, D.J. Deehan, M.J. Barter, D.A. Young

**Affiliations:** † Skeletal Research Group, Institute of Genetic Medicine, Newcastle University, Central Parkway, Newcastle Upon Tyne, NE1 3BZ, UK; ‡ Faculty of Medical Sciences, Bioinformatics Support Unit, Newcastle University, Newcastle Upon Tyne, NE2 4HH, UK; § Wellcome Trust Centre for Cell-Matrix Research, University of Manchester, Manchester, M13 9PT, UK; ‖ Freeman Hospital, Orthopaedics, Freeman Road, High Heaton, Newcastle Upon Tyne, NE7 7DN, UK

**Keywords:** Non-coding RNA, lincRNA, RNA-seq, Cartilage, Hip, Knee

## Abstract

**Objective:**

Long intergenic non-coding RNAs (lincRNAs) are emerging as key regulators in gene expression; however, little is known about the lincRNA expression changes that occur in osteoarthritis (OA). Here we aimed to define a transcriptome of lncRNAs in OA cartilage, specifically comparing the lincRNA transcriptome of knee and hip cartilage.

**Method:**

RNA-seq was performed on nucleic acid extracted from hip cartilage from patients undergoing joint replacement surgery because of either OA (*n* = 10) or because of a neck of femur fracture (NOF; *n* = 6). After transcript alignment, counts were performed using Salmon and differential expression for ENSEMBL lincRNAs determined using DESeq2. Hip RNA-seq lincRNA expression was compared to a knee dataset (ArrayExpress; E-MTAB-4304). ChIP-seq data from ENCODE was used to determine whether lincRNAs were associated with promoters (plncRNA) or unidirectional enhancer-like regulatory elements (elncRNAs).

**Results:**

Our analysis of the hip transcriptome identified 1692 expressed Transcripts Per Million (TPM ≥1) Ensembl lincRNAs, of which 198 were significantly (FDR ≤0.05) differentially expressed in OA vs normal (NOF) cartilage. Similar analysis of knee cartilage transcriptome identified 648 Emsembl lincRNAs with 93 significantly (FDR ≤0.05) differentially expressed in intact vs damaged cartilage. In total, 1834 lincRNAs were expressed in both hip and knee cartilage, with a highly significant correlation in expression between the two cartilages.

**Conclusion:**

This is the first study to use RNA-seq to map and compare the lincRNA transcriptomes of hip and knee cartilage. We propose that lincRNAs expressed selectively in cartilage, or showing differential expression in OA, will play a role in cartilage homoeostasis.

## Introduction

Osteoarthritis (OA) is a common debilitating musculoskeletal disease that affects the articulating joints and for which there remains no cure. The burden of OA continues to increase worldwide. In the United Kingdom, 89,288 hip and 98,591 knee primary replacements were recorded in the National Joint Registry in 2015, with the vast majority attributable to OA^1^. Clinically OA is characterised by pain, stiffness and deformity of the affected joint with loss of function. Macroscopically there is degradation and loss of articular cartilage with subsequent sclerosis of the underlying bone. At the molecular level, changes to chondrocytes and the composition of the extracellular matrix have been extensively investigated, with a maladaptive milieu of growth factors, inflammatory cytokines (e.g., Tumour Necrosis Factor α; TNFα) and degradative enzymes such as matrix metalloproteinases (MMPs) contributing to cartilage destruction^2^.

Gene expression changes in OA cartilage have been well characterised and indicate alterations in chondrocyte homoeostasis and activation in response to this maladaptive milieu^3^, ^4^. Long non-coding RNAs (lncRNAs) are emerging as key regulators of gene expression. They are conventionally defined as being over 200 nucleotides in length with many hallmarks of mRNA transcripts but an absence of coding ability. Many appear well conserved^5^, ^6^ though there is also evidence to the contrary^6^, ^7^ and they have been demonstrated to have tissue specificity, perhaps more so than protein-coding transcripts^8^, ^9^. Their mechanisms of action, although not fully characterised, are increasingly being recognised. They may act in *cis* or *trans* at the transcriptional and post-transcriptional level as guides or scaffolds for DNA, RNA and protein binding^10^, ^11^, manifesting – for example – in chromatin remodelling^12^. LncRNAs have key roles in development and many are proposed to have impact on disease, for example, the lncRNA HOTAIR is dysregulated in several cancers, and its overexpression has been shown to drive breast cancer metastasis^13^. Nevertheless, potential key regulatory roles for lncRNAs in both health and disease remain underexplored.

We have previously identified lncRNAs in human articular cartilage but little is known about the changes in lncRNA expression that occur in OA^14^. One recent study used RNA sequencing (RNA-seq) to look at IL-1β-induced lncRNAs in cultured chondrocytes isolated from human hip OA cartilage^15^. Other studies have used microarrays for transcriptome profiling of lncRNAs in knee OA cartilage^3^, ^4^, ^16^, and upon stimulation of isolated chondrocytes with key pro-inflammatory mediators IL-1β^4^, ^15^ and TNFα^4^, but such studies are limited by pre-existing probe design.

Whilst independent epidemiological risk factors have been established for both hip and knee OA, few studies have compared gene expression between the two. We have previously explored the gene expression profile of hip OA cartilage in comparison to age-matched neck of femur (NOF) fracture cartilage (NOF)^17^. Interestingly, when comparing our dataset to data from a similar study of knee OA cartilage we found only a small subset of differentially expressed genes in common but a large overlap in dysregulated canonical pathways. Other knee and hip OA comparison have included differential DNA methylation analysis of articular cartilage^18^, ^19^, identifying a large number of differentially methylated loci between both the joint site and disease-status.

Here we identify a repertoire of long intergenic non-coding RNAs (lincRNAs) in both hip and knee articular cartilage and the expression changes in OA progression. We highlight differential expression of lincRNAs between knee and hip to explore any pathological differences between the two at the molecular level.

## Methods

### Cartilage samples

**Fig. 1:**
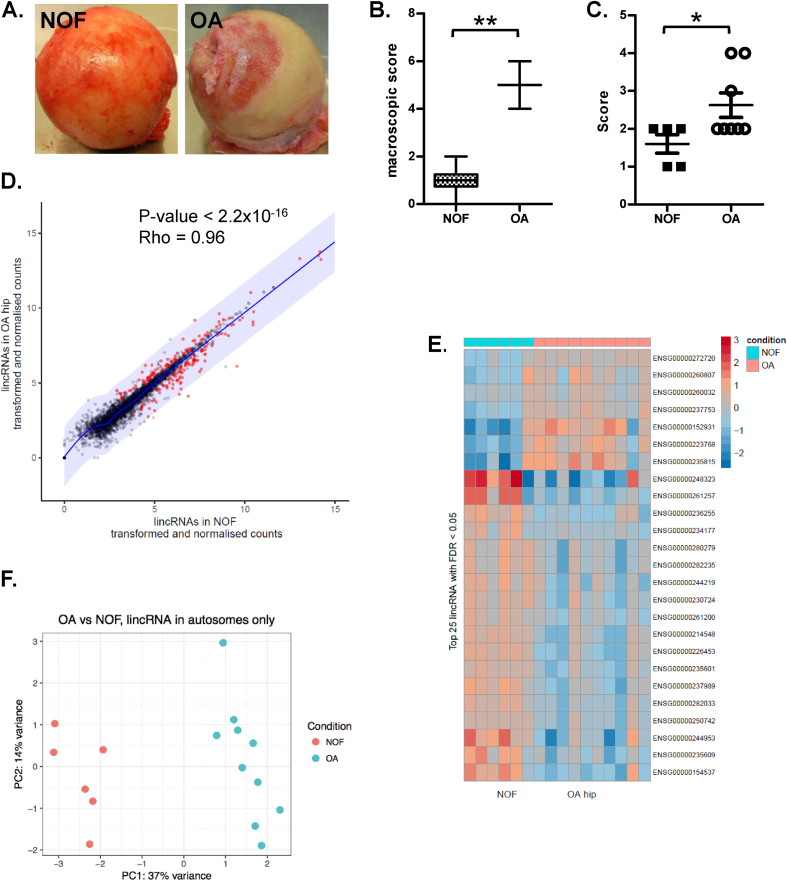
Differential lincRNA expression in NOF compared to OA hip cartilage. A. A NOF and OA femoral head used in this study. The OA femoral heads typically showed exposed bone and fibro-cartilage. B. Blinded cartilage macroscopic scores based on the Noyes classification^20^. Line mean, error bars are maximum and minimum, **represents *P* < 0.01. C. Hip osteoarthritis for NOF and OA patients were calculated from X-rays (as described in Methods) to give an overall grade (0–5) based on the Kellgren and Lawrence. Statistical significance was determined using a Mann Whitney *U* test where * *P* < 0.05. D. Scatter plot of lincRNA counts in OA vs NOF RNA-seq. Significant lincRNAs (DESeq2, FDR < 0.05) are coloured in red, the shading represents a log2 fold change of 2 from the regression line. A Spearman's rank test was used to assess the correlation between samples. E. Heatmap of top 25 differentially expressed lncRNAs in hip OA (peach) vs NOF (cyan). F. Principal component plot, hip OA vs NOF RNA-seq samples (autosomal lincRNAs only).

Human hip articular cartilage samples were obtained from consented patients undergoing joint replacement surgery due to either end-stage OA or intracapsular NOF fracture with Ethical Committee approval from the Newcastle and North Tyneside Health Authority. Joints were inspected macroscopically and scored using a scheme adapted from Noyes classification^20^ to include the presence of osteophytes [Fig. 1(A) and (B)]. Samples used in this study included those previously scored and reported^17^. In addition, radiological grading of the pre-operative X-rays was carried out by two blinded orthopaedic trainees. The Kellgren and Lawrence grading system was used to score the latest pre-operative hip X-ray for the OA group. NOF hips were scored using the trauma admission X-rays. The Kellgren and Lawrence grading system is a five-tiered system (0- no arthritis to 5- severe arthritis) that takes into account joint space narrowing, osteophytes, sclerosis, presence of cysts and bone deformity. The grading system has been validated for different joints with good inter-observer reliability^21^, ^22^. Further to the overall grade, the observers assigned a binary scoring system for the presence of each specific feature of the grading system. Data was also collected from the contralateral side in order to avoid the potential confounding factor of displacement due to the fractured NOF [Fig. 1(C)]. For all joints, macroscopically normal cartilage was collected, snap frozen in liquid nitrogen within 2 h of surgery and stored at −80°C prior to RNA extraction.

For knee, we used a pre-existing RNA-seq dataset from eight patients undergoing total knee arthroplasty for OA (age range 65–79 years, mean age 70.3; Supplementary Fig. 1(B)). The cartilage samples were from paired osteochondral specimens isolated from the ‘intact’ posterior lateral condyle (PLC) and the ‘damaged’ distal medial condyle (DMC) - sites OA^23^ (Supplementary Fig. 1(C)). To characterise the cartilage tissue, the PLC and DMC samples were graded histologically using a modified Mankin score^24^, which confirmed the DMC cartilage was significantly more damaged than that from the PLC (Supplementary Fig. 1(C) and (D)).

### RNA extraction and sequencing

For the hip RNA-seq, total RNA was isolated directly from female articular cartilage samples (OA, *n* = 10; NOF, *n* = 6) as described^25^ and RNA integrity verified as previously described^17^. mRNA was purified and 78-base paired-end sequencing performed on an Illumina Genome Analyzer IIx (Illumina Inc., Saffron Walden, UK). Sequencing reads quality controls were as described^26^. Fastq files are available to download from the National Center for Biotechnology Information Expression Omnibus GSE111358. On average each sample contained >22 million mapped read counts (see below), which is sufficient, given the number of replicates to detect differentially expressed genes with an False Discovery Rate (FDR) <0.05 with a power close to 1^27^. Knee RNA-seq data was from Dunn *et al.*^23^ ArrayExpress (E-MTAB-4304). As with the hip RNA, total knee RNA was extracted from liquid nitrogen frozen cartilage using TRIzol (LifeTechnologies) and homogenisation followed by a final purification using Qiagen RNeasy columns (Qiagen). Following sequencing on an Illumina HiSeq 2000 approximately 39 million reads per sample were generated^23^.

### RNA-seq analysis

For both knee and hip datasets, transcript counts were estimated using Salmon (version 0.7.2) in quasi-mapping mode against human reference genome hg38 (release 87) with default settings. Salmon estimated counts were summarized to gene level using the tximport package in RStudio for use with DESeq2. The RUVg *in silico* approach was used to remove unwanted technical variation. RUVg variation factors were added into the design model of DESeq2 for differential expression testing. Differential expression analysis was performed paired and unpaired for OA knee and hip, respectively. All genes were included in DESeq2 analysis before filtering for genes designated as a “lincRNA” biotype by ENSEMBL. Heatmaps were generated with log2 transformed and normalised counts using the pheatmap() function in RStudio.

To generate scatterplots, the removeBatchEffect() function from limma was used with the DESeq2 normalised and log2 transformed counts, giving the RUVg unwanted variation factors as a co-variate. The ggplot2 package was used in Rstudio to build the scatterplots.

To generate genome browser tracks, reads were aligned to hg38 using the HISAT2 aligner. Alignment files were converted into bigwig format first by converting into bedgraph format using BEDTools genomecov and then the bedGraphToBigWig utility from UCSC.

### Enhancer and promoter lincRNA derivation

Histone ChIP-seq read alignment tracks for H3K4me3, H3K4me1 and H3K27ac for Roadmap cell type E049 (bone marrow-derived chondrocytes) accessed from NCBI GEO database, accession GSE19465. Bed tracks were converted to hg38 using the UCSC liftover tool and converted to bam format using the bedtobam utility in bedtools. Sorted bam files were used as input into ngs.plot.r^28^ to plot read coverage around the transcription start sites (TSS) of lincRNAs (±2000 bp). LincRNAs with a mean Transcripts Per Million (TPM) > 1 in all hip and knee samples were included. H3K4me1 and H3K4me3 coverages were plotted as bam pairs. The gene order was extracted using R from H3K4me1/H3K4me3 heatmaps and used to plot H3K27ac read coverage.

## Results

For hip, we performed RNA-seq on cartilage RNA extracted from OA and NOF patients undergoing total hip arthroplasty. The NOF samples had a significantly lower level of OA than the OA samples, both macroscopically^17^ and by X-ray analysis [Fig. 1(A)–(C)]. There was no significant difference in the ages of the consented patients in each grouping (Supplementary Fig. 1(A)). RNA-seq analysis of all hip cartilage samples identified 1692 Ensembl lincRNAs with an average TPM ≥1. Ensembl lincRNA classification is based on cDNA alignments overlapping chromatin methylation regions (H3K4me3 or H3K36me3) outside of protein-coding loci. LincRNAs (long intergenic noncoding RNAs) are better described as long intervening non-coding RNAs and analysis of the this portion of lncRNAs avoids the complication arising from overlap with coding genes^7^.

When stratifying these hip cartilage samples into OA and NOF, DESeq2 analysis identified 198 lincRNAs as differentially expressed (FDR ≤0.05) with 28 of these showing a fold change ≥2 (log2 fold ≥1) (Fig. 1(D) and (E); Supplementary Table 1). Of these 198 and 28, 89 and 7 were increased, respectively, in OA hip cartilage compared to NOF. Importantly, when using just the expression data from these 1692 lincRNAs principal component analysis (PCA) segregated the OA and NOF samples, suggesting lincRNA expression alone is enough to define disease severity [Fig. 1(F)]. The most upregulated lincRNAs in hip OA included PART1, LINC01139 and NORAD, while the most downregulated included LUCAT1, MEG3 and LINC01679. LincRNAs have been proposed to regulate local gene expression^29^ but we could find no correlation between OA severity-associated changes in lincRNA expression with those of the neighbouring protein-coding genes (data not shown).

**Fig. 2:**
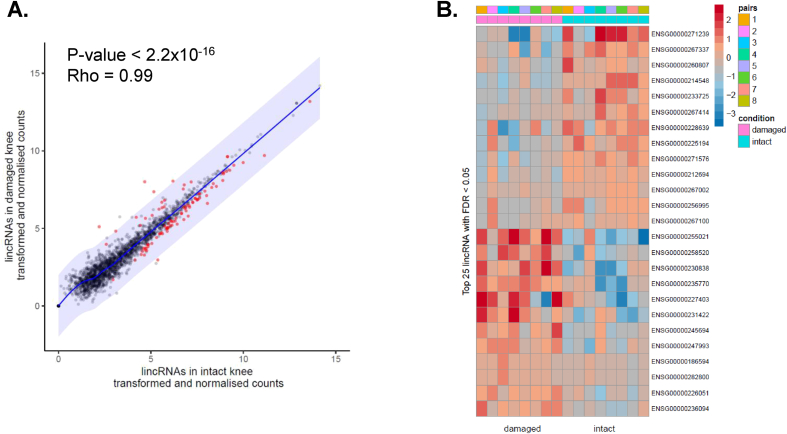
Differential lincRNA expression in paired OA-intact (PLC) compared to OA-damaged DMC) knee cartilage. A. Scatter plot of lincRNA counts in OA knee intact vs damaged RNA-seq. Significant lincRNAs (DESeq2, FDR < 0.05) are coloured in red, the shading represents a log2 fold change of 2 from the regression line. A Spearmans rank test was used to assess the correlation between samples. B. Heatmap of top 25 differentially expressed lncRNAs in damaged (pink) vs intact (cyan) knee OA RNA-seq samples. Patient pairings are indicated (1–8).

Next we performed a similar RNA-seq analysis pipeline for a previously published knee OA RNA-seq dataset^23^. Cartilage for the knee study was taken from eight patients and contained paired osteochondral samples isolated from both the intact PLC and the damaged DMC for each donor (Supplementary Fig. 1(C) and (D)). This analysis identified a total of 648 Ensembl lincRNAs with a TPM ≥1 in all damaged DMC and intact PLC OA knee cartilage samples. In the knee data, 93 lincRNAs were differentially expressed (FDR ≤0.05) between the damaged (DMC) and intact (PLC) cartilage groups with 13 of these showing a fold change ≥2 (log2 fold ≥1) (Fig. 2(A) and (B); Supplementary Table 2). Similarly, of these 93 and 13, 20 and 8 respectively were upregulated in the damaged knee cartilage, the remainder downregulated. The most upregulated lincRNAs in damaged knee cartilage were CRNDE, MIR22HG and LINC01614. The most downregulated lincRNAs included MEG3, ILF3-AS1 and LINC01089.

**Fig. 3:**
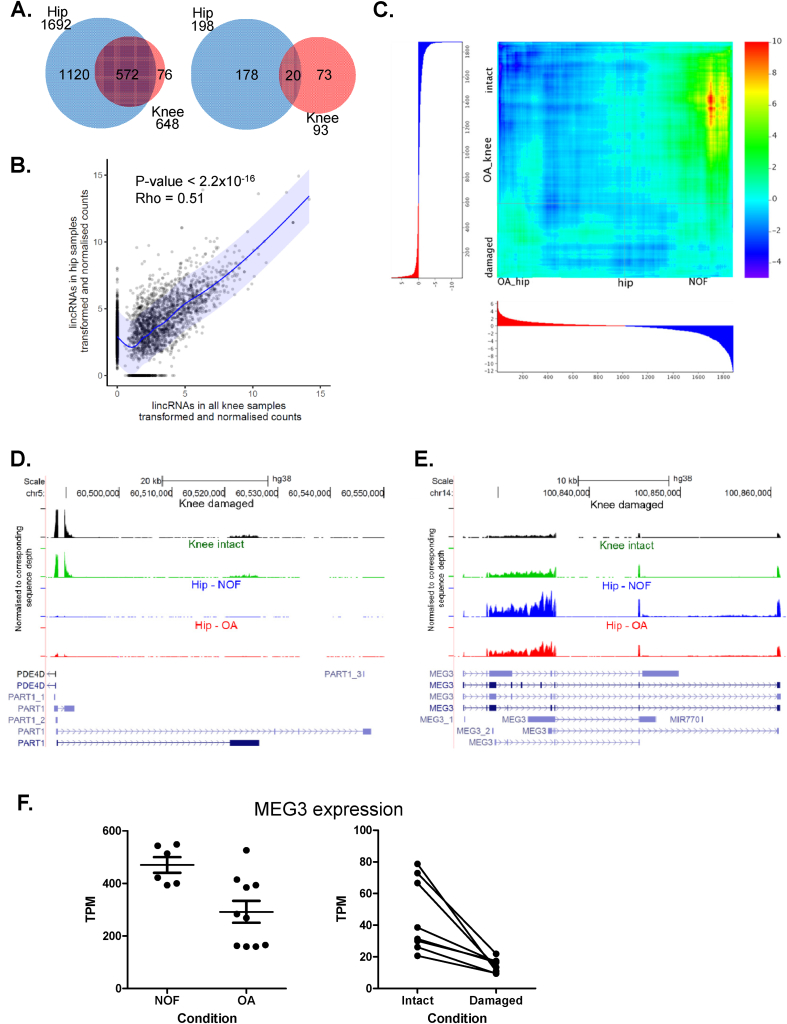
Joint site comparisons in lincRNA expression. A. Venn diagrams showing the 572 lincRNAs in common between hip and knee and those differentially expressed between the tissues and disease status at both joint sites (20). B. Scatter plot of mean lincRNA counts (TPM) for all hip samples (NOF and OA hip) vs all knee (intact and damaged) samples. C. Rank–rank hypergeometric overlap representation of lincRNA lists ranked by the degree of differential expression observed in the two profiling experiments (positive values indicate an enrichment of overlapping lincRNAs). Key depicts –log10 *P* value. Axis graphs depict ranked gene regulation with red and blue highlighting upregulated and downregulated lincRNAs, respectively, in damaged knee or OA hip datasets. D. Total read coverages of each sample group on UCSC Browser hg38. PART1 expression in damaged & intact knee OA, and hip OA & NOF. E. Read coverage plot on UCSC Browser hg38. MEG3 expression in damaged & intact knee OA, and hip OA & NOF. F. MEG3 expression in individual NOF vs hip OA and intact vs damaged paired knee OA samples. TPM for each experimental dataset are presented.

When comparing lincRNAs between hip and knee 572 were detected in common between the cartilages from both joints [Fig. 3(A)]. Furthermore, the average expression level of all lincRNAs between the two cartilages showed a good degree of correlation [Fig. 3(B)], especially when considering the known variability of lincRNA expression levels between tissues^7^. Of the differentially expressed lincRNAs from hip or knee only 20 lincRNAs were in common including established lincRNAs such as NEAT1 and MEG3 (Table I). However, when using the whole lincRNA dataset for each joint ranked by degree of differential expression using the Rank–rank Hypergeometric Overlap (RRHO) threshold-free algorithm^30^ a robust correlation was observed in lincRNAs with reduced expression in intact knee and NOF cartilage [Fig. 3(C)]. Of course, one important caveat is that neither dataset contains a true non-OA control.

**Table I:** Differentially expressed LincRNAs in common between OA hip vs NOF and intact vs damage knee cartilage

Ensembl ID	lincRNA name	Hip_Av_TPM	Knee_Av TPM	Hip log2 Fold Change	Knee log2 Fold Change	Hip adjusted pvalue	Knee adjusted pvalue
ENSG00000260807	RP11-161M6.2	73.26	21.41	0.669	0.692	3.17E-04	3.05E-06
ENSG00000230724	LINC01001	40.41	4.61	−0.735	0.659	4.91E-04	6.60E-03
ENSG00000214548	MEG3	381.13	30.04	−0.864	1.350	1.25E-03	4.23E-11
ENSG00000261200	RP11-989E6.10	5.91	0.55	−0.629	0.719	1.43E-03	1.25E-02
ENSG00000235815	RP1-125N5.2	8.95	9.94	1.220	0.684	1.56E-03	3.14E-03
ENSG00000230606	AC159540.1	32.76	14.93	0.504	0.646	1.72E-03	5.21E-03
ENSG00000179523	EIF3J-AS1	21.25	17.74	−0.405	0.322	2.01E-03	6.31E-03
ENSG00000212694	LINC01089	20.08	14.49	0.421	0.792	2.21E-03	3.01E-06
ENSG00000272374	RP3-329A5.8	0.61	0.23	−0.746	0.849	3.07E-03	1.29E-02
ENSG00000203709	C1orf132	8.65	0.65	−1.058	0.462	3.28E-03	1.88E-02
ENSG00000224652	LINC00885	0.99	0.94	0.887	0.713	5.05E-03	8.52E-03
ENSG00000230487	PSMG3-AS1	4.77	2.51	0.625	0.504	7.08E-03	7.18E-03
ENSG00000245532	NEAT1	1149.80	250.31	−0.865	0.407	9.60E-03	4.22E-02
ENSG00000255135	RP11-111M22.3	11.10	12.76	−0.389	0.390	1.20E-02	4.41E-02
ENSG00000232931	LINC00342	6.02	2.93	0.284	0.566	2.93E-02	2.41E-02
ENSG00000283445	RP11-63G10.4	1.90	0.79	0.406	0.758	2.99E-02	2.84E-03
ENSG00000261888	AC144831.1	1.34	1.14	−0.599	−0.727	3.00E-02	2.63E-03
ENSG00000255284	AP006621.5	4.79	4.29	0.451	0.531	3.39E-02	4.87E-02
ENSG00000265485	RP11-449D8.1	2.07	4.83	0.638	0.419	4.93E-02	3.64E-02
ENSG00000256321	RP11-153K16.1	3.02	2.05	−0.496	0.794	4.99E-02	3.83E-03

Further to identifying the lincRNAs altered during OA progression and cartilage damage we also sought to determine whether any lincRNAs exhibit joint site selective expression levels. Initially we examined the relative expression of all coding transcripts (after removing the mitochondrial encoded genes) between the differing cartilage joint sites. Correlation analysis of the expression level of all coding mRNAs gave a Spearman's Rank of 0.87 (*P* < 0.00001) (Supplementary Fig. 1). 18 of the 25 most abundant mRNAs were common between the two joint sites, with the remainder found in the top 100 expressed transcripts, the only exception being superoxide dismutase 3 (SOD3) which was expressed higher in hip. Patterning differences between the two joint sites were reflected by differential expression of HOX genes with more 3′ HOX cluster genes (HOXA4-6 and HOXB1-3) showing higher expression in hip, with more 5′ genes (HOXD8-10) enriched in knee – possibly demonstrating maintenance of the temporal sequential expression pattern observed during development^31^. Correlation analysis of the expression level of all the lincRNAs gave a Spearman's Rank of 0.51 (*P* < 0.00001) [Fig. 3(B)]. Of the 10 most abundant lincRNAs in each cartilage type, 8 were in common, with the remainder being found in the top 100 expressed lincRNAs. Some lincRNA showed joint site differential expression. For example, in knee cartilage the lincRNA PART1 was more abundant than in hip, yet was only significantly upregulated in OA hip cartilage vs NOF [Fig. 3(D)]. On the contrary MEG3 was more abundant in hip compared to knee cartilage [Fig. 3(E)] but differentially expressed in both [Fig. 3(E) and (F)].

**Fig. 4:**
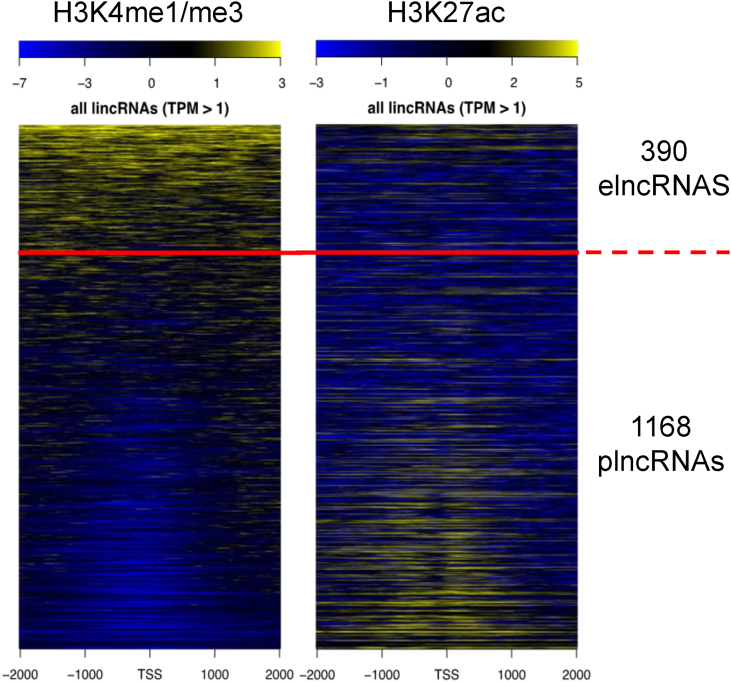
Histone ChIP-seq analysis of chondrocyte lincRNA. Histone ChIP-seq read alignment tracks for H3K4me3, H3K4me1 and H3K27ac were analysed from Roadmap cell type E049 (bone marrow-derived chondrocytes). Data presented are from the 1558 lincRNAs with a mean TPM > 1 in all hip and knee samples. Read coverage density is plotted around the TSS of lincRNAs (±2000 bp). H3K4me1 and H3K4me3 coverages were plotted as bam pairs using ngs.plot, with the same lincRNA order used to subsequently plot the H3K27ac read coverage. To define enhancer-associated (elncRNA) and promoter-associated (plncRNA) lincRNAs a threshold of 0.5 of H3K4me1/H3K4me3 was defined (red line).

Numerous studies have identified a large number of lincRNAs, far outstripping the number of coding transcripts. However, the bottleneck in research has been in determining which lincRNAs are functional. Several studies have attempted to address how to identify functional lincRNAs. We used a modification of the method defined by Marques *et al.*^32^ which divides lincRNAs as either promoter–associated lncRNAs (plncRNA) or unidirectional enhancer-like regulatory element associated lncRNAs (elncRNAs) based on their overlying chromatin status. plncRNAs and elncRNAs are defined as those with an enriched H3K4me1 or H3K4me3 mark respectively at or around their TSS. We used histone ChIP-seq data from bone marrow derived chondrocytes generated as part of Roadmap^33^ to define the H3K4Me1 to H3K4Me3 ratio around (+/− 2 kbp) the TSS of the lincRNAs expressed in both hip and knee cartilage (Fig. 4). We also included H3K27Ac as a marker of biological activity. Approximately 75% of these cartilage lincRNAs were defined as plncRNAs; those which have been proposed have a function beyond regulation of the nearest gene^32^. plncRNAs had a slightly higher, but non-significant, expression levels compared to elncRNAs (plncRNA mean TPM of 6.82 and 6.35 for hip and knee respectively vs an elncRNA mean TPM of 4.37 for hip and knee respectively). plncRNAs included PART1 and MEG3.

## Discussion

Current non-coding RNA research in OA has generally focused on microRNAs. Here for the first time we have used RNA sequencing to define lincRNA expression in OA in both hip and knee cartilage. We identified 198 lincRNAs altered in hip OA progression and 93 in damaged knee cartilage of which 20 were in common. We additionally identified lincRNAs with joint site selectivity and further assessed their potential functionality by interrogating their chromatin status. Pearson *et al.*, have also used RNA-Seq to profile lncRNAs in chondrocytes, although their focus was on those lncRNAs upregulated by IL-1^15^. In contrast to our RNA extracted from cartilage, their study makes use of isolated chondrocytes. It is known that chondrocytes that are isolated dedifferentiate, adopting a more fibroblastic phenotype^34^, ^35^, ^36^. The authors also derived some of their non-OA cartilage from post-mortem donors, but only for real-time PCR analysis of a subset of IL-1-induced lncRNAs. Conversely the hip and knee OA samples used for RNA-seq in our study were obtained from live tissue donors and processed within 2 h post-surgery.

Interestingly, lincRNAs can be detected within exosomes present in extra-cellular fluid such as serum and therefore could represent biomarkers of disease status, as suggested for cancer^37^. Recently for OA, exosomal levels of lncRNA PCGEM1 in synovial fluid has been proposed as a biomarker for different stages of disease^38^.

Importantly, we have highlighted lincRNAs that are differentially expressed in damaged OA cartilage. Of interest, we observed that the lincRNA ROCR (ENSG00000228639), which we identified and characterised as a critical regulator of MSC chondrogenesis, is significantly down-regulated in damaged knee cartilage, but not differentially expressed in the hip dataset^14^, ^39^. Further, our results show that MEG3 is significantly downregulated in both OA hip and damaged knee OA cartilage, confirming knee cartilage data from Su *et al.*^40^ and data from a rodent OA-model^41^. However, this somewhat contrasts with the findings of Fu and colleagues where MEG3 transcript isoforms were both down or upregulated in knee OA cartilage compared to age-matched non-OA controls^3^. MEG3 has been principally identified as an influential tumour suppressor, whereby it negatively regulates cell proliferation and induces apoptosis^42^, ^43^, ^44^, ^45^. Downregulation of this imprinted lincRNA has been associated with multiple cancers^43^, ^44^, ^45^, ^46^, ^47^. In a rat OA model MEG3 has been proposed to regulate miR-16 expression, thus indirectly regulating Smad7^41^. Indeed, others have shown that MEG3 may negatively regulate the Transforming growth factor (TGF)β signalling pathway^48^, with isolated fibroblasts overexpressing MEG3 unable to proliferate when incubated with TGFβ^49^. TGFβ plays a somewhat pleiotropic role in both normal and OA joints. In normal tissue it is active only transiently following joint loading while in OA joints high TGFβ activity is detected^50^. MEG3 may additionally have a role in the osteogenic differentiation of MSCs. Zhuang *et al.*, examined MEG3 and BMP4 expression in isolated MSCs. In these cells the overexpression of MEG3 resulted in greater expression of osteogenic markers such as RUNX2, whilst reducing MEG3 expression had the opposite effect^51^.

We have also highlighted lincRNAs that may be selective for knee (e.g., PART1) and hip (e.g., MEG3), respectively. Interestingly, though many lincRNAs were shared between the two joints only 20 lincRNAs were differentially expressed in common between the disease states of both joint sites. When we previously used microarray analysis to explore gene expression changes in normal and OA cartilage of hip and knee, we similarly found a limited overlap (approximately 10%) of differentially expressed genes^17^. The two joints in question do have independent risk factors for OA such as gender, ethnicity and occupation^52^, ^53^, ^54^, the molecular and pathophysiological basis of which remain elusive. Further, although highly correlative, the two joint sites do have characteristic gene expression programmes, potentially regulated by differential Hox gene expression^31^. Interestingly, the Hox gene cluster is well known to include a number of well-studied non-coding transcripts^55^. Of particular interest, lncRNA-HIT appears a critical regulator of early limb development^56^.

The challenge remains to determine which of the lincRNAs we and others have identified are functional and which lack a specific biological function. Although lincRNA loci may contain active promoters and associated chromatin marks and the transcripts themselves can be capped, spliced and polyadenylated, none of these features offer an informative indicator of function. Marques *et al.*, describe a multifaceted approach to discerning the biological relevance of lincRNAs^32^. Using their criteria, we classified the cartilage expressed lincRNAs into plncRNAs and elncRNAs, with the majority in our analysis being plncRNAs. The association of plncRNAs with their own promoter suggests a potentially more evolutionary conserved role than their elncRNA counterparts. Testing the function of lincRNAs still remains a challenge. Gene function is often initially sought using experimental animals, most commonly the genetically tractable mouse. However, determining the function of a lncRNA by its deletion in mice can also be fraught since removing the genetic loci can also disrupt important underlying DNA elements such as enhancers^57^. Moreover, using comparative genomics to understand the function of lincRNAs is not straightforward since the majority show little sequence or splicing pattern conservation. There are notable exceptions, but these tend to be those lncRNAs that are relatively highly expressed^58^. With these caveats in mind, much recent work has described interactions between short and lncRNAs^59^. In OA, several lncRNAs have been shown act as potential ‘sponges’ to regulate microRNA function^60^, ^61^.

In summary, we have outlined lincRNA expression patterns in both hip and knee OA cartilage. We have highlighted differential expression between hip OA and NOF and between intact and damaged knee OA (sites of low and high mechanical loading, respectively). We have additionally highlighted significantly changing lincRNAs that are common to both, namely the tumour suppressor MEG3, which is downregulated. Mondal *et al.* identified MEG3 as forming DNA-RNA triplex complexes with chromatin, targeting constituents of the TGFβ signalling pathway; indeed^48^, fully characterising the mechanisms of action of lincRNAs in general remains another key area for development.

## Declaration of contributions

DAY and MJB were involved in concept and study design. BA, KC, YX, AP, AJS, MJB and DAY were involved in collection, assembly and analysis and interpretation of the data. YX and MJB processed patient material. KC and AJS also provided statistical expertise. DJD was involved in the provision of study materials/patient samples. JS and TEH provided the knee data, histology and patient demographic data. DAY, MJB and DJD obtained the funding. All authors were involved in drafting the manuscript and gave final approval of the version submitted.

## Competing interest statement

All authors declare no competing interests.
