# Supplementary material for: Identification of long non-coding RNAs expressed in knee and hip osteoarthritic cartilage
Source: Osteoarthritis Cartilage. 2019 Apr;27(4):694–702. doi: 10.1016/j.joca.2018.12.015 (PMC6444060; doi:10.1016/j.joca.2018.12.015)
Supplement: Multimedia component 1 [file mmc1.pdf]

Supplementary Figure 1

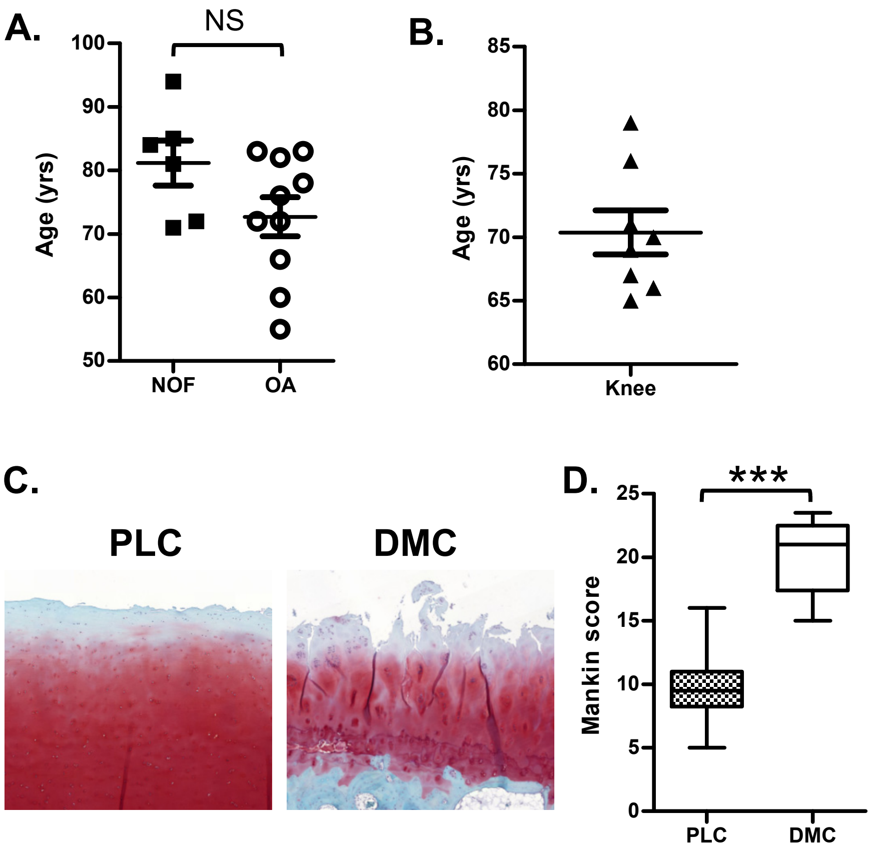

A. Age (in years) of patients at time of arthroplasty. Ten OA hip samples are on average younger than the NOF (mean OA 72.7 years old; NOF 81.2 yo), however using a variety of statistical tests these are not significantly different. OA, are patients undergoing arthroplasty due to hip osteoarththritis; NOF, are patients undergoing arthroplasty due to a neck of femur fracture. **B.** Age of the donors of the knee cartilage specimens. The age of the patients was not significantly different from the hip OA patients. Box plots include a line for the mean, error bars are maximum and minimum. **C.** Histologcal comparison from paired (knee) osteochondral samples exemplifying the undamaged (PLC - intact posterior lateral condyle and damaged (DMC - distal medial condyle). Osteochondral blocks were processed, sectioned and stained as decribed in Dunn et al., (2016). **D.** The PLC and DMC OA samples were graded histologically using a modified Mankin score (Little et al., 2010). The DMC samples showed significantly more damaged cartilage compared to the PLC (Mann Whitney u test, *** $p<0.001$).
