# Supplementary material for: Identification of long non-coding RNAs expressed in knee and hip osteoarthritic cartilage
Source: Osteoarthritis Cartilage. 2019 Apr;27(4):694–702. doi: 10.1016/j.joca.2018.12.015 (PMC6444060; doi:10.1016/j.joca.2018.12.015)
Supplement: Multimedia component 2 [file mmc2.pdf]

Supplementary Figure 2

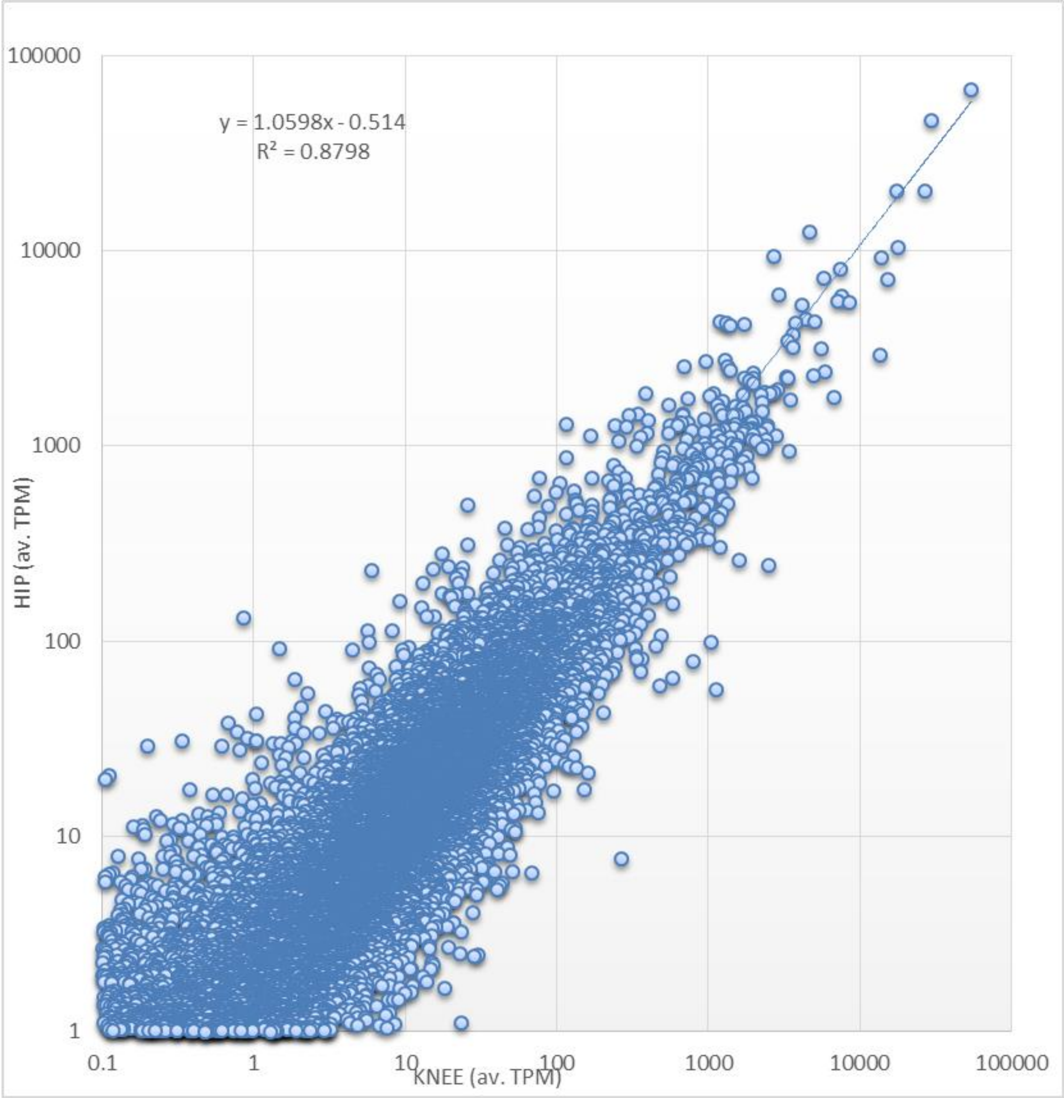

Correlation of expression level (average TPM) between protein-coding genes expressed in common between hip and knee cartilage. In total 12625 transcripts with an expression level TPM ≥1 were expressed in both conditions. The data were significantly correlated ($p<0.00001$).
